# Advantages of a novel *in situ* pH measurement for soilless media

**DOI:** 10.3389/fpls.2024.1334328

**Published:** 2024-03-27

**Authors:** Noah James Langenfeld, Hikari Ai Skabelund, Royal Heins, Bruce Bugbee

**Affiliations:** ^1^ Crop Physiology Laboratory, Utah State University, Logan, UT, United States; ^2^ Department of Horticultural Sciences, Texas A&M University, College Station, TX, United States

**Keywords:** pH electrode, controlled environments, peat moss, coconut coir, pine bark, perlite, saturated paste, pour-through

## Abstract

Rhizosphere pH determines nutrient bioavailability, but this pH is difficult to measure. Standard pH tests require adding water to growth media. This dilutes hydrogen ion activity and increases pH. We used a novel, *in situ*, pointed-tip electrode to estimate rhizosphere pH without dilution. Measurements from this electrode matched a research-grade pH meter in hydroponic nutrient solutions. We then compared measurements from this electrode to saturated paste and pour-through methods in peat moss, coconut coir, and pine bark. The pointed-tip electrode was unable to accurately measure pH in the highly-porous pine bark media. Adding deionized water to the other media at container capacity using the saturated paste method resulted in a pH that was 0.59 ± 0.30 units higher than the initial *in situ* measurement at the top of the container. This increase aligns with established solution chemistry principles. Measurements of pH using the pour-through method were 0.38 ± 0.24 pH units higher than *in situ* measurements at the bottom of the container. We conclude that *in situ* pH measurements are not subject to dilution and are thus more representative of the rhizosphere pH than the saturated paste and pour-through techniques.

## 1 Introduction

Nutrient bioavailability depends on rhizosphere pH, which is the pH directly adjacent to root surfaces. The rhizosphere pH is difficult to measure and is often estimated from pH measurements of the bulk substrate. Smiley (1974) found that the rhizosphere pH varied from the bulk pH by 1.2 pH units for wheat (*Triticum aestivum*) plants depending on the nitrogen source (nitrate or ammonium). Nye (1981) further modelled that the bulk pH could vary from the rhizosphere pH by 1 to 2 pH units depending on distance from the root surface and nitrogen source. Methods to better estimate rhizosphere pH from bulk pH are needed.

Ion exchange principles are the same between field soils and soilless media substrates. The lower bulk density and higher water holding capacity of soilless media for horticultural crops often increases growth compared to field soils for agronomic crops. Container-grown plants can have more pH management challenges and experience more rapid pH changes than field-grown plants due to the confined root-zone with reduced media buffering capacity.

The saturated paste method is widely used to measure the bulk pH of field soils and container media (Miller and Kissel, 2010; Thomas, 2018). A substrate sample is removed from the container, deionized water is added until it is saturated and visibly glistens, and the pH of the saturated paste is measured (Kalra, 1995). Alternatively, the media solution may be vacuum-extracted and subsequently measured with a pH electrode. Media to water dilutions of 1:2 and 1:5 are also commonly used to determine bulk pH.

The pour-through method measures the pH of container leachate and does not require removal of the media (Wright, 1986). A tray is placed beneath the container and water, or a fertigation solution, is slowly poured through the container to collect a liquid sample in the tray (generally 50 to 100 mL). Yao et al. (2008) found no significant effect on pH when the leachate volume ranged from 40 mL to 120 mL in sphagnum moss planted with moth orchids (*Phalaenopsis* spp.). Torres et al. (2010) found no consistent differences between the pour-through pH of a 50 mL leachate and a 2.5% leachate of a peat moss, perlite, and bark mix planted with boxwood (*Buxus* x *koreana*). Small leachate volumes may not capture a representative pH, especially if root-zone stratifications exist. Altland and Owen (2024) analyzed the pH of pine bark media that had been intentionally stratified between fertilizer-amended and non-amended fractions within a container. They concluded that pH measurements using the pour-through method were always more similar to the pH measurement of a 1:1 saturated paste extraction from the bottom half of the container, regardless of stratification or particle size.

Saturated paste and pour-through methods have been compared in several studies. Yeage et al. (1983), Wright et al. (1990), and Cavins et al. (2008) found no difference in pH using saturated paste or pour-through methods with distilled water. A recent review of several studies indicated minimal differences between saturated paste and pour-through methods (Altland, 2021). However, both approaches require dilution of the root-zone solution.

The addition of deionized water to nutrient solutions dilutes hydrogen ion activity and increases pH. Sumner (1994) found an increase of 0.5 pH units when comparing saturated paste measurements to *in situ* measurements in soils. Schofield and Taylor (1955) reduced the dilution effect by measuring soil pH in a solution of 0.01 M calcium chloride. Matthiesen (2004) reported that saturated paste measurements were higher than *in situ* measurements by up to 0.4 pH units when using a pointed-tip pH electrode for pH measurements of soils, but the addition of calcium chloride to the samples minimized this increase. The dilution effect therefore increases the error in estimating rhizosphere pH.

The *in situ* method utilizes direct insertion of a pH electrode into the media and is faster than other methods. The electrode tip must contact the media solution, but electrodes with pointed tips facilitate contact with the media. *In situ* pH measurements remove the dilution effect experienced from saturated paste and pour-through pH measurements. There is no rhizosphere in unplanted containers, so the dilution effect simply makes bulk pH measurements inaccurate. The *in situ* method could help researchers and growers more closely estimate pH values and nutrient availabilities for plants grown in soilless media.

No previous studies have compared these pH measurement methods in soilless media – this was the objective of our novel work. We hypothesized that *in situ* measurements would be lower than saturated paste and pour-through measurements due to the absence of the dilution effect.

## 2 Methods

### 2.1 pH electrode

A pointed-tip pH electrode (model HALO2 GroLine, Hanna Instruments, Woonsocket, RI, USA) was used to measure pH in all parts of the study (**Supplementary Figure 1A**). Preliminary studies (not shown) concluded identical measurements between the pointed-tip electrode and a research-grade electrode, which was evidence for its accuracy. The pH meter was paired with the Hanna Lab app on a smartphone through Bluetooth® for calibration. The gelled electrolyte inside the electrode needed to be refilled after about every 200 measurements.

### 2.2 Media types and composition

**Figure 1:**
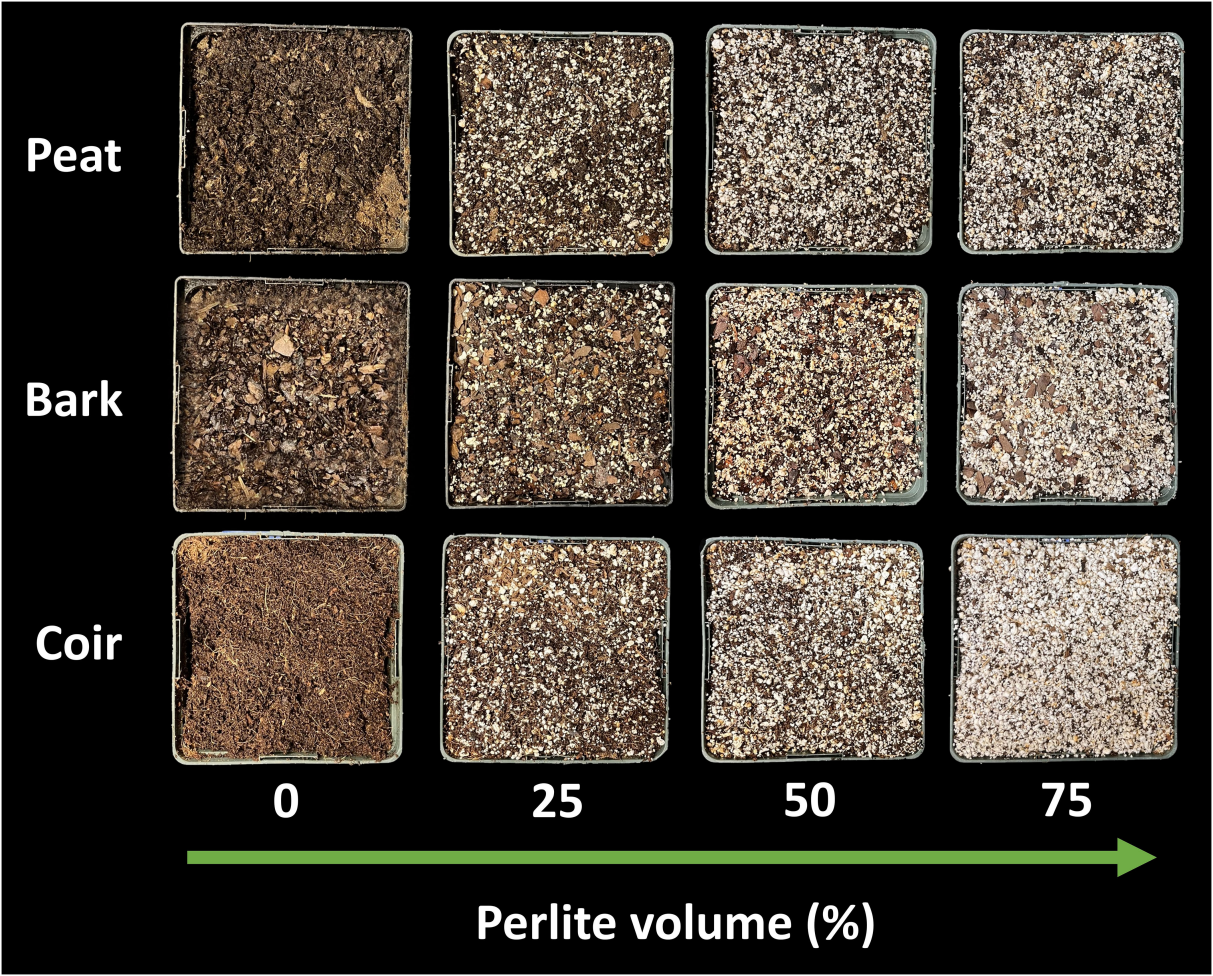
Peat moss, pine bark, and coconut coir mixed with increasing levels of perlite (0% to 75% by volume).

Containers with a volume of 1.7-L were filled with one of three media types: peat moss (Premier Pro-Moss TBK; Premier Horticulture, Inc., Quakertown, PA, USA), coconut coir (Black Gold Just Coir; Sun Gro Horticulture, Agawam, MA, USA), or pine bark (from *Pinus taeda*, particle size less than 2 cm). Each media type was then amended with 0%, 25%, 50%, or 75% perlite (Expanded Perlite; Hess Pumice, Malad City, ID, USA) by volume (**Figure 1**). Wetting agent (AquaGro® 2000 G; Aquatrols, Paulsboro, NJ, USA) was added at one gram per liter of media. Hydrated lime (calcium hydroxide) was added as needed to adjust pH of the peat from pH 5 to 7. Lime was not added to coconut coir or pine bark. Each treatment included three replicate containers. The containers were then saturated to container capacity with a nutrient solution containing 120 ppm nitrogen (72 ppm nitrate nitrogen, 42 ppm ammonium nitrogen, and 6 ppm urea nitrogen), 31 ppm phosphorus, 177 ppm potassium, 45 ppm calcium, 19 ppm magnesium, 25 ppm sulfur, 17 ppm silicon, 0.9 ppm iron, 0.3 ppm manganese, 0.3 ppm zinc, 0.41 ppm boron, 0.85 ppm copper, and 0.06 ppm molybdenum. The nutrient solution pH was 6.7 and electrical conductivity was 1.4 mS per cm. Containers drained for 1 hour before making pour-through and saturated paste pH measurements.

### 2.3 Dilution effect on pH

The pH of the above nutrient solution was measured after diluting with deionized water from a dilution factor of 1 to 10. This was repeated with a nutrient solution without phosphorus (P) to represent root-zone conditions with low buffering capacity (P concentrations are typically low in the root-zone as P is actively taken up by plants). This amended nutrient solution contained 84 ppm nitrogen, 141 ppm potassium, 60 ppm calcium, 19 ppm magnesium, 26 ppm sulfur, 17 ppm silicon, 0.39 ppm iron, 0.16 ppm manganese, 0.2 ppm zinc, 0.43 ppm boron, 0.25 ppm copper, 0.001 ppm molybdenum and 0.0003 ppm nickel.

### 2.4 Pour-through method

Pour-through measurements were undertaken first to ensure all replicates were near container capacity. The 1.7-L containers were slowly watered with 1 L of the nutrient solution described in the previous section to minimize channeling of the solution through the container. The leachate was collected in a tray and pH was measured. The 1 L leachate volume was selected to ensure displacement of the root-zone solution while not being large enough to contribute to the displaced solution.

### 2.5 Moist vs. wet *in situ* measurements

Moist *in situ* measurements were made in three locations on the media at the top of the container following pour-through measurements (water content just below container capacity). The pH meter was inserted at a depth of 4 cm and a reading was recorded after stabilization (about 5 s, **Supplementary Figure 1B**). A wet *in situ* measurement was then taken in the same location after adding three to five mL of deionized water onto the area and reinserting the probe. Bottom *in situ* measurements were made 4 cm deep into three locations at the bottom of the container through drainage holes.

### 2.6 Saturated paste method

The pH was measured by removing media around the three locations previously sampled on the top of the container to loosely fill a 30-mL beaker. Deionized water (electrical conductivity of less than 0.005 mS per cm) was added to the beaker to create a saturated paste as in Kalra (1995). The pH was then measured directly in the beaker using the *in situ* pH meter.

### 2.7 Unplanted vs. planted media

The replicate media measured in the above tests were then seeded with lettuce (*Lactuca sativa* cv. Grand Rapids). The same tests were repeated in these planted containers 28 days after seeding.

### 2.8 Statistical analysis

Measurement and planting methods were compared with T-tests and ANOVA using RStudio (Posit Software, PBC, Boston, MA). Replicate measurements using the same method in the same container were averaged to eliminate pseudo replicates.

## 3 Results

### 3.1 pH dilution from deionized water

**Figure 2:**
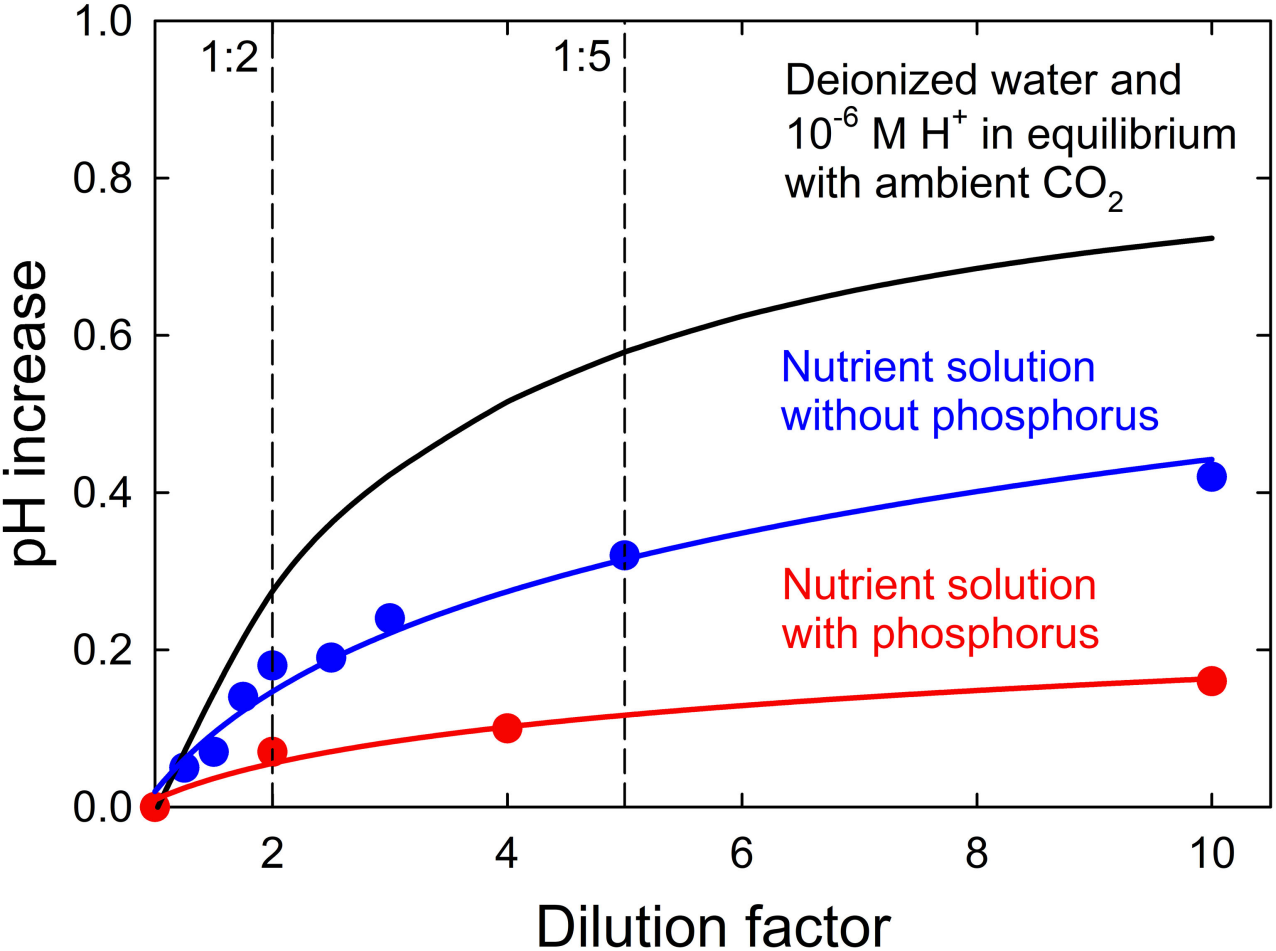
The pH increase of a solution with deionized water and 10^-6^ M hydrogen ions in equilibrium with ambient CO_2_ (3.4 x 10^-4^ atm), a nutrient solution without phosphorus, and a nutrient solution with phosphorus as the dilution factor with deionized water increases from 1 to 10.

The pH increased 0.16 pH units as the dilution factor of the complete nutrient solution increased from 1 to 10 (**Figure 2**). Removing phosphorus from the nutrient solution increased the pH 0.42 units as the dilution factor increased from 1 to 10. The pH would be expected to increase 0.72 units with only deionized water and hydrogen ions in equilibrium with ambient CO_2_ (3.4 x 10^-4^ atm; 400 ppm CO_2_ at 86 kPa atm) as the dilution factor increased from 1 to 10. Dissolved carbon dioxide produces carbonic acid, which buffers the solution and reduces the dilution effect.

### 3.2 Pre-wetting the measurement area

Wetting the measurement area with deionized water prior to pH measurement (wet *in situ*) did not change the pH (**Supplementary Figure 2**) if the media moisture content was greater than 3 on a 5-point moisture scale (Huang and Fisher, 2013). Measurements were erratic if the media was at moisture level 1 or 2 on the scale (data not shown).

### 3.3 Comparing *in situ* with saturated paste and pour-through

**Figure 3:**
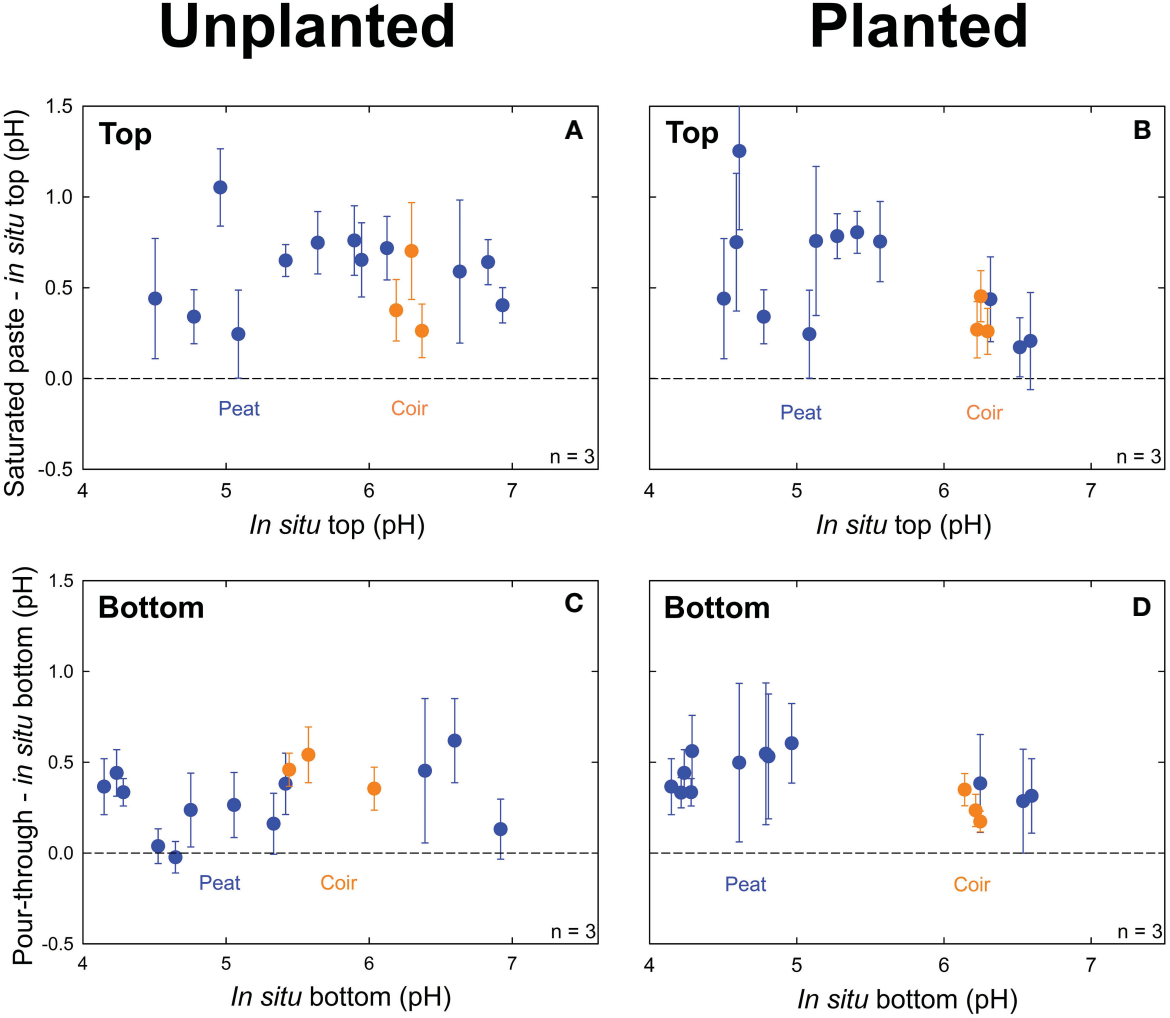
pH measurements in unplanted containers and containers planted with lettuce (*Lactuca sativa*). The average difference between pH measurements from the saturated paste and *in situ* methods at the top of the container versus *in situ* pH at the top of the container in unplanted containers **(A)** and planted containers **(B)**. The difference between pH measurements from the pour-through and *in situ* pH methods at the bottom of the container versus *in situ* pH at the bottom of the container in unplanted containers **(C)** and planted containers **(D)**. Peat moss or coconut coir were mixed with 0%, 25%, or 50% perlite to obtain 15 treatments with 3 containers per treatment. Error bars represent standard deviation of the container measurements.

Saturated paste pH averaged 0.57 ± 0.22 (p = 0.05, n = 30) pH units higher than top *in situ* pH in unplanted containers (**Figure 3A**) and 0.61 ± 0.33 (p = 0.02, n = 30) pH units higher in planted containers (**Figure 3B**). Pour-through pH averaged 0.31 ± 0.18 (p = 0.36, n = 30) pH units higher than bottom *in situ* pH in unplanted containers (**Figure 3C**) and 0.45 ± 0.19 pH (p = 0.22, n = 30) units higher in planted containers (**Figure 3D**). Treatments with pine bark and 75% perlite-based media had highly variable *in situ* pH measurements with reduced plant growth from low water retention. They were not included in the above data and were not analyzed further.

### 3.4 Saturated paste vs. pour-through pH

**Figure 4:**
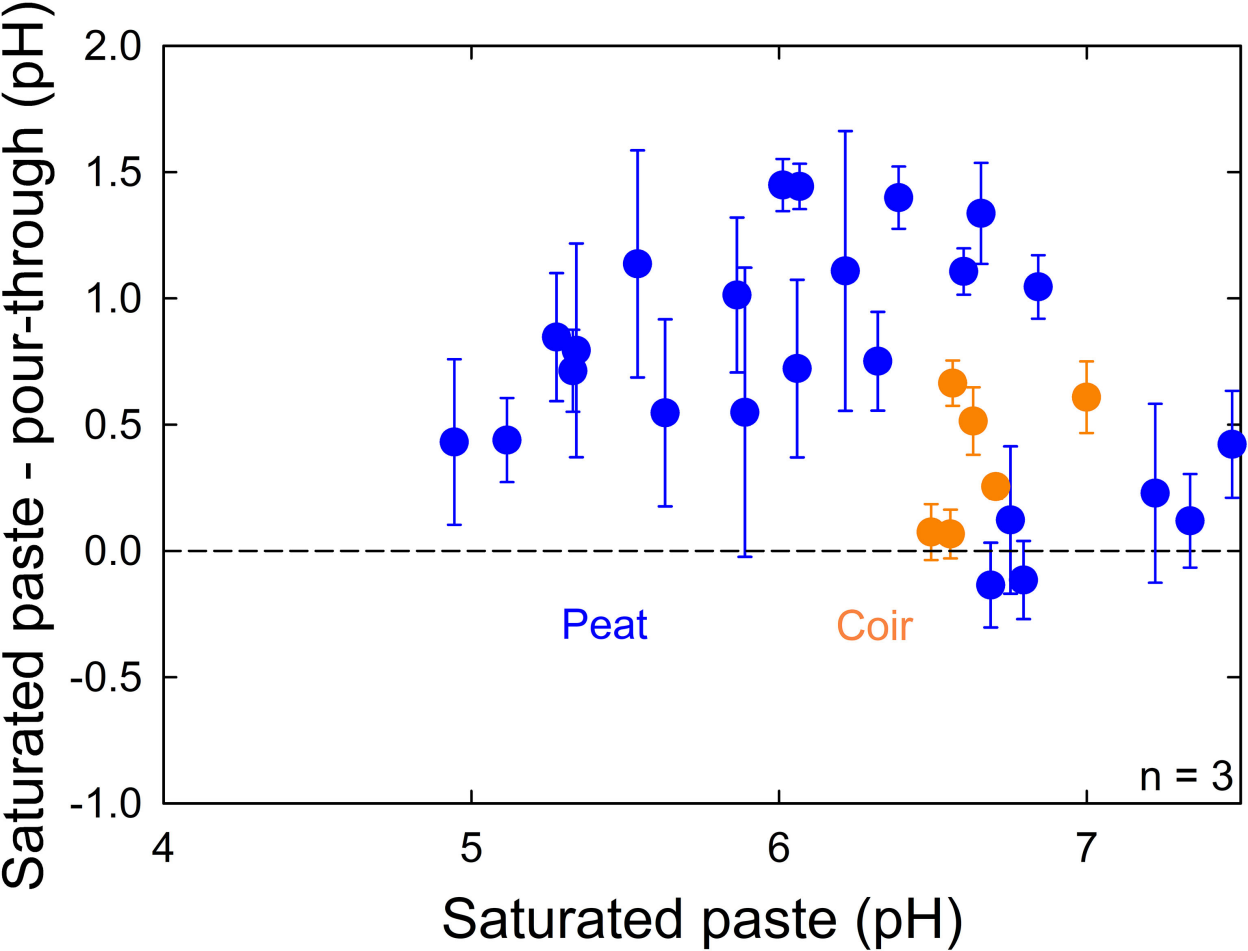
The difference between saturated paste and pour-through pH measurements compared to saturated paste pH measurements in both unplanted containers and containers planted with lettuce (*Lactuca sativa*). The difference between saturated paste and pour-through measurements was significant (p = 0.003), but there was no significant difference between unplanted and planted containers (p = 0.54). Peat moss or coconut coir were mixed with 0%, 25%, or 50% perlite to obtain 15 treatments with 3 containers per treatment. Error bars represent standard deviation of the container measurements.

We found saturated paste pH measurements at the top of the container to be significantly higher (0.66 ± 0.47, p = 0.003, n = 60) than pour-through pH measurements (**Figure 4**), but there was no significant difference between unplanted and planted containers (p = 0.54, n = 60).

### 3.5 pH meter stability

The meters we used (n = 2) displayed stabilized measurements within 5 seconds throughout their more than 20 months of use. The only periodic maintenance that was required was refilling the electrolyte gel after about every 200 samples and removing humus materials using an acidic cleaning solution from the manufacturer every few months.

## 4 Discussion

Similar to our results, Matthiesen (2004) found a deviation of *in situ* pH compared to saturated paste pH in wet soils up to 1 pH unit. Keaton (1938) measured the pH of soil samples from across the United States using increasing ratios of water to soil. He demonstrated a general increase in pH for soils as the water to soil ratio increased. His measurements were 0.5 pH units higher for a 1:1 dilution and 0.9 pH units higher for a 10:1 dilution than for samples at the original soil moisture content, which is in a similar range to our results. Keaton hypothesized this discrepancy was due to cation exchange and differential base saturation, which he confirmed by observing little change in pH measurements among soil moistures when soils were completely desaturated of metal ions.

Increasing the dilution factor from *in situ* to saturated paste measurements reduces H^+^ activity and increases pH (Davis, 1943; Peech, 1965). In a pure solution, dilution increases pH in acidic solutions and decreases pH in alkaline solutions. However, in soil or media suspensions, pH always increases with dilution. The increasing base saturation in alkaline suspensions (Havlin, 2005) leads to base cation hydrolysis with increasing dilution, which buffers out any expected decrease in pH (Thomas, 2018) and continues to make it susceptible to the dilution effect (Conyers and Davey, 1988). Keaton (1938) demonstrated that the pH increased from 6.6 to 7.5 in alkaline soils when diluting 1:1. Higher dilutions can also increase the mobility of potassium ions from pH electrodes leading to higher pH readings (Sumner, 1994).

The dilution effect can often be minimized by fixing the ionic strength using calcium chloride. Miller and Kissel (2010) found that the pH measured with the saturated paste method using water was consistently about 0.5 pH units higher than when measured with dilute calcium chloride. Sumner (1994) additionally found that measurement in calcium chloride reduced pH measurement variability. Although using calcium chloride can counteract the effect of ion activity, it is an additional step, and was not analyzed in this paper.

The significantly higher pH measurements of the saturated paste method compared to the pour-through method that we found are in contrast to the results of Altland (2021) and Cavins et al. (2008) discussed earlier. The media, lime, and nutrient solution may not have been at equilibrium in unplanted pots measured after 2 h, but we found the same discrepancy in planted pots after 28 days. This suggests that this effect is independent of stage in the crop life cycle. While Yeage et al. (1983), Wright et al. (1990), and Cavins et al. (2008) used distilled water in the pour-through method, we used nutrient solution, which could have displaced more H^+^ ions and decreased pH. Our leachate volume was higher than others, but we observed no bias in diluting pH with increasing leachate volumes.

We did not include treatments that had stunted and variable lettuce growth among replicates. This occurred with pine bark and treatments with 75% perlite by volume. The pine bark had larger particle sizes and was more hydrophobic than the peat moss and coconut coir even after addition of the wetting agent. These characteristics made moisture retention more difficult in pine bark leading to uneven moisture distribution and variable *in situ* pH measurements. The high air-filled porosity with 75% perlite similarly led to variable *in situ* measurements. Pour-through pH measurements were more consistent than the *in situ* measurements in these circumstances as they captured larger areas.

We selected a common pH range from 5 to 7 because metal toxicities, such as those from aluminium (Imadi et al., 2016) and manganese (El-Jaoual and Cox, 1998), are more common below pH 5 and iron precipitates and becomes unavailable above pH 7 (Colombo et al., 2014). The peat moss, coconut coir, and pine bark were representative of media components used in research and commercial production. The peat moss was more acidic (Elliott, 1996) than coconut coir and pine bark and required the addition of lime to increase the pH.

Increased solution contact from increased media moisture should lead to increased pH measurement accuracy, but we observed no difference in pH measurements between moist (no pre-wetting, moisture level 3 to 4) and wet (pre-wetting, moisture level 4) *in situ* insertion techniques. This was likely because we only added 3 to 5 mL of deionized water and the media we tested was already near container capacity. We observed increasing measurement variability in preliminary studies when the media was visually dry (moisture level 1 to 2). This was presumably because contact between the electrode and media surface was incomplete.

## 5 Conclusion

Our measurements of the pH of soilless media using the saturated paste and pour-through methods were consistently 0.4 to 0.6 pH units higher than *in situ* measurements. Measuring pH using saturated paste and pour-through methods dilutes hydrogen ion activity and results in a higher pH that may not be representative of rhizosphere conditions.

We found no bias between peat moss or coconut coir media, but *in situ* measurements were more variable in media with pine bark and perlite levels of 75%.

*In situ* measurements are not subject to dilution effects experienced by saturated paste and pour-through measurements and may provide a more accurate indication of rhizosphere pH.

## Data availability statement

The original contributions presented in the study are included in the article/**Supplementary Material**. Further inquiries can be directed to the corresponding author.

## Author contributions

NL: Conceptualization, Data curation, Formal analysis, Investigation, Methodology, Software, Validation, Visualization, Writing – original draft, Writing – review & editing. HS: Data curation, Formal analysis, Investigation, Methodology, Software, Validation, Visualization, Writing – original draft, Writing – review & editing. RH: Conceptualization, Writing – review & editing. BB: Conceptualization, Funding acquisition, Project administration, Resources, Supervision, Writing – review & editing.
