# Supplementary material for: Advantages of a novel in situ pH measurement for soilless media
Source: Front Plant Sci. 2024 Mar 27;15:1334328. doi: 10.3389/fpls.2024.1334328 (PMC11004321; doi:10.3389/fpls.2024.1334328)
Supplement: Supplementary file 1 [file DataSheet_1.docx]

Advantages of *in situ* pH measurement in soilless media

Supplementary Material

# Supplementary Data

**Supplementary Table 1.** The increase in pH with dilution factor for nutrient solutions with and without phosphorus.

| **Nutrient solution with phosphorus** | | |  | **Nutrient solution without phosphorus** | | |
| --- | --- | --- | --- | --- | --- | --- |
| Dilution Factor | pH | pH increase |  | Dilution Factor | pH | pH increase |
| 1 | 6.73 | 0 |  | 1 | 6.26 | 0 |
| 2 | 6.80 | 0.07 |  | 1.25 | 6.31 | 0.05 |
| 4 | 6.83 | 0.10 |  | 1.50 | 6.33 | 0.07 |
| 10 | 6.89 | 0.16 |  | 1.75 | 6.40 | 0.14 |
|  |  |  |  | 2 | 6.44 | 0.18 |
|  |  |  |  | 2.5 | 6.45 | 0.19 |
|  |  |  |  | 3 | 6.50 | 0.24 |
|  |  |  |  | 5 | 6.58 | 0.32 |
|  |  |  |  | 10 | 6.68 | 0.42 |

**Supplementary Table 2.** *In situ* pH measurements using moist and wet insertions of the meter in unplanted peat moss (data for supplemental figure 2).

| **Moist insertion** |  | **Wet insertion** |
| --- | --- | --- |
| 4.49 |  | 4.55 |
| 4.17 |  | 4.40 |
| 4.27 |  | 4.39 |
| 4.40 |  | 4.48 |
| 4.34 |  | 4.44 |
| 4.49 |  | 4.62 |
| 4.60 |  | 4.46 |
| 4.23 |  | 4.25 |
| 4.17 |  | 4.20 |
| 4.96 |  | 4.72 |
| 5.58 |  | 5.59 |
| 4.91 |  | 4.73 |
| 5.09 |  | 5.15 |
| 4.52 |  | 4.77 |
| 5.18 |  | 4.83 |
| 5.20 |  | 5.12 |
| 5.31 |  | 5.34 |
| 5.03 |  | 5.19 |
| 5.96 |  | 5.63 |
| 4.65 |  | 4.45 |
| 6.11 |  | 5.79 |
| 5.94 |  | 5.85 |
| 4.92 |  | 5.10 |
| 6.40 |  | 6.37 |
| 6.14 |  | 6.17 |
| 6.87 |  | 7.02 |
| 7.20 |  | 7.49 |
| 7.65 |  | 7.59 |
| 6.26 |  | 6.07 |
| 7.27 |  | 7.31 |
| 6.83 |  | 6.76 |
| 6.48 |  | 6.66 |
| 7.62 |  | 7.18 |

**Supplementary Table 3**. The pH of unplanted peat moss containers (n = 3) with increasing perlite volumes using the pour-through, saturated paste, top *in situ*, and bottom *in situ* methods.

| **Perlite (%)** | **Pour-through** | **Saturated paste** | | |  | **Top *in situ*** | | |  | **Bottom *in situ*** | | |
| --- | --- | --- | --- | --- | --- | --- | --- | --- | --- | --- | --- | --- |
| 0 | 4.68 | 4.87 | 4.76 | 5.63 |  | 4.21 | 4.28 | 4.63 |  | 4.15 | 4.13 | 4.14 |
|  | 4.52 | 4.95 | 4.87 | 4.53 |  | 5.18 | 4.38 | 4.09 |  | 4.21 | 4.11 | 4.14 |
|  | 4.34 | 4.73 | 5.21 | 4.95 |  | 4.48 | 4.89 | 4.40 |  | 4.14 | 4.14 | 4.17 |
| 25 | 4.49 | 4.95 | 5.11 | 5.18 |  | 4.71 | 4.91 | 4.73 |  | 4.21 | 4.24 | 4.20 |
|  | 4.81 | 5.09 | 5.14 | 5.21 |  | 4.87 | 4.63 | 4.76 |  | 4.28 | 4.25 | 4.26 |
|  | 4.73 | 4.98 | 5.03 | 5.35 |  | 4.78 | 4.80 | 4.79 |  | 4.25 | 4.21 | 4.22 |
| 50 | 4.65 | 5.38 | 5.25 | 5.37 |  | 5.30 | 4.98 | 5.08 |  | 4.27 | 4.25 | 4.29 |
|  | 4.52 | 5.39 | 5.39 | 5.31 |  | 4.97 | 5.18 | 5.25 |  | 4.31 | 4.29 | 4.25 |
|  | 4.68 | 5.03 | 5.49 | 5.36 |  | 5.23 | 4.89 | 4.89 |  | 4.30 | 4.28 | 4.30 |
| 75 | 5.80 | 5.96 | 5.85 | 5.84 |  | 5.22 | 5.46 | 5.18 |  | 4.78 | 4.51 | 4.77 |
|  | 5.83 | 5.72 | 6.09 | 6.13 |  | 5.26 | 5.62 | 5.48 |  | 4.78 | 4.53 | 4.72 |
|  | 5.76 | 5.95 | 5.20 | 5.88 |  | 5.71 | 4.87 | 5.53 |  | 5.09 | 4.60 | 4.63 |

**Supplementary Table 4.** The average and standard deviation of pH in unplanted peat moss containers (n = 3) with increasing perlite volumes using the pour-through, saturated paste top *in situ*, and bottom *in situ* methods.

| **Perlite (%)** | **Pour-through** | |  | **Saturated paste** | |  | **Top *in situ*** | |  | **Bottom *in situ*** | |
| --- | --- | --- | --- | --- | --- | --- | --- | --- | --- | --- | --- |
|  | **Avg.** | **S.Dev** |  | **Avg.** | **S.Dev** |  | **Avg.** | **S.Dev** |  | **Avg.** | **S.Dev** |
| 0 | 4.51 | 0.17 |  | 4.94 | 0.32 |  | 4.50 | 0.35 |  | 4.15 | 0.03 |
| 25 | 4.68 | 0.17 |  | 5.12 | 0.12 |  | 4.78 | 0.08 |  | 4.24 | 0.03 |
| 75 | 4.62 | 0.09 |  | 5.33 | 0.13 |  | 5.09 | 0.16 |  | 4.28 | 0.02 |
| 100 | 5.80 | 0.04 |  | 5.85 | 0.27 |  | 5.37 | 0.26 |  | 4.71 | 0.18 |

**Supplementary Table 5.** The average differences and standard deviations among pH measurement methods in unplanted peat moss with increasing perlite volume.

| **Perlite (%)** | **Difference (pH)** | **Average** | **Standard Deviation** |
| --- | --- | --- | --- |
| 0 | Sat. Paste – Top *in situ* | 0.44 | 0.33 |
|  | Pour-through – Bottom *in situ* | 0.37 | 0.15 |
|  | Sat. paste – Pour-through | 0.43 | 0.33 |
| 25 | Sat. Paste – Top *in situ* | 0.34 | 0.15 |
|  | Pour-through – Bottom *in situ* | 0.44 | 0.13 |
|  | Sat. paste – Pour-through | 0.44 | 0.17 |
| 50 | Sat. Paste – Top *in situ* | 0.24 | 0.24 |
|  | Pour-through – Bottom *in situ* | 0.33 | 0.08 |
|  | Sat. paste – Pour-through | 0.71 | 0.16 |
| 75 | Sat. Paste – Top *in situ* | 0.48 | 0.17 |
|  | Pour-through – Bottom *in situ* | 1.08 | 0.19 |
|  | Sat. paste – Pour-through | 0.05 | 0.26 |

**Supplementary Table 6**. The pH of unplanted peat moss with lime amendment of 0.4 g hydrated lime per kg (n = 3) with increasing perlite volumes using the pour-through, saturated paste top *in situ*, and bottom *in situ* methods.

| **Perlite (%)** | **Pour-through** | **Saturated paste** | | |  | **Top *in situ*** | | |  | **Bottom *in situ*** | | |
| --- | --- | --- | --- | --- | --- | --- | --- | --- | --- | --- | --- | --- |
| 0 | 4.58 | 6.04 | 5.90 | 6.01 |  | 5.38 | 5.05 | 5.01 |  | 4.45 | 4.65 | 4.51 |
|  | 4.60 | 6.14 | 6.09 | 5.92 |  | 5.03 | 4.88 | 4.85 |  | 4.39 | 4.51 | 4.64 |
|  | 4.51 | 5.97 | 5.89 | 6.15 |  | 4.95 | 4.75 | 4.74 |  | 4.58 | 4.52 | 4.48 |
| 25 | 4.63 | 6.15 | 5.91 | 6.08 |  | 5.42 | 5.37 | 5.39 |  | 4.69 | 4.67 | 4.76 |
|  | 4.60 | 6.13 | 6.04 | 6.01 |  | 5.42 | 5.54 | 5.37 |  | 4.49 | 4.75 | 4.66 |
|  | 4.64 | 6.16 | 5.97 | 6.15 |  | 5.45 | 5.38 | 5.41 |  | 4.59 | 4.60 | 4.61 |
| 50 | 5.04 | 6.51 | 6.49 | 6.65 |  | 5.93 | 5.74 | 5.83 |  | 5.07 | 4.92 | 4.63 |
|  | 5.01 | 6.36 | 6.21 | 6.35 |  | 5.72 | 5.67 | 5.52 |  | 4.67 | 4.92 | 4.76 |
|  | 4.92 | 6.28 | 6.43 | 6.22 |  | 5.58 | 5.31 | 5.47 |  | 4.36 | 4.53 | 4.92 |
| 75 | 6.15 | 6.33 | 6.41 | 6.57 |  | 5.94 | 6.06 | 6.03 |  | 5.42 | 5.25 | 5.15 |
|  | 5.88 | 6.40 | 6.41 | 6.51 |  | 5.47 | 5.93 | 5.71 |  | 4.97 | 5.11 | 4.83 |
|  | 5.97 | 6.38 | 6.36 | 6.35 |  | 5.68 | 5.35 | 5.69 |  | 5.00 | 4.99 | 4.96 |

**Supplementary Table 7**. The average and standard deviation of pH in unplanted peat moss with lime amendment of 0.4 g hydrated lime per kg containers (n = 3) with increasing perlite volumes using the pour-through, saturated paste top *in situ*, and bottom *in situ* methods.

| **Perlite (%)** | **Pour-through** | |  | **Saturated paste** | |  | **Top insertion** | |  | **Bottom insertion** | |
| --- | --- | --- | --- | --- | --- | --- | --- | --- | --- | --- | --- |
|  | **Avg.** | **S.Dev** |  | **Avg.** | **S.Dev** |  | **Avg.** | **S.Dev** |  | **Avg.** | **S.Dev** |
| 0 | 4.56 | 0.05 |  | 6.01 | 0.10 |  | 4.96 | 0.19 |  | 4.53 | 0.09 |
| 25 | 4.62 | 0.02 |  | 6.07 | 0.09 |  | 5.42 | 0.05 |  | 4.65 | 0.08 |
| 75 | 4.99 | 0.06 |  | 6.39 | 0.15 |  | 5.64 | 0.19 |  | 4.75 | 0.22 |
| 100 | 6.00 | 0.14 |  | 6.41 | 0.08 |  | 5.76 | 0.25 |  | 5.08 | 0.18 |

**Supplementary Table 8.** The difference in multiple methods of measuring pH values in unplanted peat with lime amendment of 0.4 g hydrated lime per kg (n = 3) at increasing levels of perlite.

| **Perlite (%)** | **Difference (pH)** | **Average** | **Standard Deviation** |
| --- | --- | --- | --- |
| 0 | Sat. Paste – Top *in situ* | 1.05 | -0.22 |
|  | Pour-through – Bottom *in situ* | 0.04 | 0.10 |
|  | Sat. paste – Pour-through | 1.45 | 0.10 |
| 25 | Sat. Paste – Top *in situ* | 0.65 | 0.09 |
|  | Pour-through – Bottom *in situ* | -0.02 | 0.09 |
|  | Sat. paste – Pour-through | 1.44 | 0.09 |
| 50 | Sat. Paste – Top *in situ* | 0.75 | 0.17 |
|  | Pour-through – Bottom *in situ* | 0.24 | 0.20 |
|  | Sat. paste – Pour-through | 1.40 | 0.12 |
| 75 | Sat. Paste – Top *in situ* | 0.65 | 0.23 |
|  | Pour-through – Bottom *in situ* | 0.92 | 0.11 |
|  | Sat. paste – Pour-through | 0.41 | 0.14 |

**Supplementary Table 9**. The pH of unplanted peat moss with lime amendment of 0.9 g hydrated lime per L (n = 3) with increasing perlite volumes using the pour-through, saturated paste top *in situ*, and bottom *in situ* methods.

| **Perlite (%)** | **Pour-through** | **Saturated paste** | | |  | **Top *in situ*** | | |  | **Bottom *in situ*** | | |
| --- | --- | --- | --- | --- | --- | --- | --- | --- | --- | --- | --- | --- |
| 0 | 5.43 | 6.66 | 6.71 | 6.64 |  | 6.04 | 6.02 | 6.03 |  | 5.50 | 5.18 | 5.14 |
|  | 5.26 | 6.60 | 6.41 | 6.70 |  | 5.83 | 5.87 | 5.58 |  | 5.11 | 4.76 | 5.06 |
|  | 5.27 | 6.47 | 7.08 | 6.64 |  | 5.78 | 6.07 | 5.85 |  | 4.90 | 4.77 | 5.08 |
| 25 | 5.52 | 6.49 | 6.65 | 6.64 |  | 5.73 | 5.86 | 6.64 |  | 5.66 | 5.57 | 5.30 |
|  | 5.51 | 6.49 | 6.62 | 6.56 |  | 6.05 | 6.00 | 6.21 |  | 5.29 | 5.15 | 5.45 |
|  | 5.45 | 6.64 | 6.62 | 6.69 |  | 6.01 | 6.15 | 5.79 |  | 5.23 | 5.20 | 5.14 |
| 50 | 5.95 | 7.29 | 6.88 | 6.94 |  | 6.35 | 5.95 | 6.11 |  | 5.22 | 5.45 | 5.43 |
|  | 5.72 | 6.84 | 6.80 | 6.73 |  | 6.23 | 5.95 | 6.02 |  | 5.45 | 5.46 | 5.39 |
|  | 5.72 | 6.66 | 6.71 | 6.73 |  | 6.15 | 6.20 | 6.16 |  | 5.37 | 5.51 | 5.46 |
| 75 | 6.43 | 6.80 | 6.81 | 6.99 |  | 6.27 | 6.23 | 6.36 |  | 5.94 | 6.05 | 6.13 |
|  | 6.37 | 7.00 | 6.96 | 6.89 |  | 6.31 | 6.43 | 6.57 |  | 6.10 | 6.23 | 5.86 |
|  | 6.48 | 6.94 | 6.90 | 7.03 |  | 6.00 | 6.23 | 6.20 |  | 6.37 | 6.20 | 6.21 |

**Supplementary Table 10**. The average and standard deviation of pH in unplanted peat moss with lime amendment of 0.9 g hydrated lime per L containers (n = 3) with increasing perlite volumes using the pour-through, saturated paste top *in situ*, and bottom *in situ* methods.

| **Perlite (%)** | **Pour-through** | |  | **Saturated paste** | |  | **Top insertion** | |  | **Bottom insertion** | |
| --- | --- | --- | --- | --- | --- | --- | --- | --- | --- | --- | --- |
|  | **Avg.** | **S.Dev** |  | **Avg.** | **S.Dev** |  | **Avg.** | **S.Dev** |  | **Avg.** | **S.Dev** |
| 0 | 5.32 | 0.10 |  | 6.66 | 0.19 |  | 5.90 | 0.16 |  | 5.06 | 0.23 |
| 25 | 5.49 | 0.04 |  | 6.60 | 0.07 |  | 5.95 | 0.18 |  | 5.33 | 0.19 |
| 75 | 5.80 | 0.13 |  | 6.84 | 0.19 |  | 6.12 | 0.13 |  | 5.42 | 0.08 |
| 100 | 6.43 | 0.06 |  | 6.92 | 0.08 |  | 6.29 | 0.16 |  | 6.12 | 0.16 |

**Supplementary Table 11.** The difference in multiple methods of measuring pH values in unplanted peat with lime amendment of 0.9 g hydrated lime per L (n = 3) at increasing levels of perlite.

| **Perlite (%)** | **Difference (pH)** | **Average** | **Standard Deviation** |
| --- | --- | --- | --- |
| 0 | Sat. Paste – Top *in situ* | 0.76 | 0.19 |
|  | Pour-through – Bottom *in situ* | 0.26 | 0.18 |
|  | Sat. paste – Pour-through | 1.34 | 0.20 |
| 25 | Sat. Paste – Top *in situ* | 0.65 | 0.20 |
|  | Pour-through – Bottom *in situ* | 0.16 | 0.17 |
|  | Sat. paste – Pour-through | 1.11 | 0.09 |
| 50 | Sat. Paste – Top *in situ* | 0.72 | 0.17 |
|  | Pour-through – Bottom *in situ* | 0.38 | 0.17 |
|  | Sat. paste – Pour-through | 1.05 | 0.13 |
| 75 | Sat. Paste – Top *in situ* | 0.64 | 0.18 |
|  | Pour-through – Bottom *in situ* | 0.31 | 0.14 |
|  | Sat. paste – Pour-through | 0.50 | 0.09 |

**Supplementary Table 12**. The pH of unplanted peat with lime amendment of 4.4 g hydrated lime per L (n = 3) with increasing perlite volumes using the pour-through, saturated paste top *in situ*, and bottom *in situ* methods.

| **Perlite (%)** | **Pour-through** | **Saturated paste** | | |  | **Top *in situ*** | | |  | **Bottom *in situ*** | | |
| --- | --- | --- | --- | --- | --- | --- | --- | --- | --- | --- | --- | --- |
| 0 | 6.72 | 8.61 | 7.40 | 7.11 |  | 7.11 | 6.92 | 6.33 |  | 6.66 | 6.34 | 6.88 |
|  | 6.99 | 7.15 | 6.62 | 6.73 |  | 6.73 | 6.19 | 6.49 |  | 6.09 | 6.21 | 5.97 |
|  | 6.82 | 7.29 | 7.01 | 7.08 |  | 6.49 | 6.63 | 6.81 |  | 6.26 | 6.66 | 6.44 |
| 25 | 7.06 | 7.53 | 7.34 | 7.26 |  | 7.08 | 6.92 | 6.81 |  | 6.67 | 6.51 | 6.69 |
|  | 7.37 | 7.25 | 7.50 | 7.28 |  | 7.02 | 7.18 | 6.76 |  | 6.60 | 6.77 | 6.78 |
|  | 7.22 | 7.28 | 7.36 | 7.22 |  | 6.97 | 6.94 | 6.71 |  | 6.65 | 6.07 | 6.64 |
| 50 | 7.18 | 7.41 | 7.52 | 7.45 |  | 6.92 | 6.64 | 6.71 |  | 7.06 | 7.06 | 7.20 |
|  | 7.11 | 7.37 | 7.58 | 7.33 |  | 6.73 | 6.96 | 6.87 |  | 6.81 | 6.76 | 6.79 |
|  | 6.86 | 7.37 | 7.63 | 7.59 |  | 6.73 | 6.97 | 6.95 |  | 6.85 | 6.99 | 6.75 |
| 75 | 7.35 | 7.36 | 7.38 | 7.27 |  | 6.74 | 6.92 | 7.00 |  | 6.95 | 6.58 | 7.27 |
|  | 7.27 | 7.48 | 7.42 | 7.50 |  | 7.01 | 6.90 | 6.97 |  | 7.01 | 7.04 | 6.83 |
|  | 7.34 | 7.36 | 7.27 | 7.28 |  | 6.89 | 6.23 | 7.02 |  | 7.12 | 6.97 | 6.94 |

**Supplementary Table 13**. The average and standard deviation of pH in unplanted peat with lime amendment of 4.4 g hydrated lime per L (n = 3) with increasing perlite volumes using the pour-through, saturated paste top *in situ*, and bottom *in situ* methods.

| **Perlite (%)** | **Pour-through** | |  | **Saturated paste** | |  | **Top insertion** | |  | **Bottom insertion** | |
| --- | --- | --- | --- | --- | --- | --- | --- | --- | --- | --- | --- |
|  | **Avg.** | **S.Dev** |  | **Avg.** | **S.Dev** |  | **Avg.** | **S.Dev** |  | **Avg.** | **S.Dev** |
| 0 | 6.84 | 0.14 |  | 7.22 | 0.58 |  | 6.63 | 0.29 |  | 6.39 | 0.30 |
| 25 | 7.22 | 0.16 |  | 7.34 | 0.11 |  | 6.93 | 0.15 |  | 6.60 | 0.21 |
| 75 | 7.05 | 0.17 |  | 7.47 | 0.11 |  | 6.83 | 0.13 |  | 6.92 | 0.16 |
| 100 | 7.32 | 0.04 |  | 7.37 | 0.09 |  | 6.85 | 0.25 |  | 6.97 | 0.19 |

**Supplementary Table 14.** The average differences and standard deviations among pH measurement methods in unplanted peat with lime amendment of 4.4 g hydrated lime per L (n = 3) with increasing perlite volumes.

| **Perlite (%)** | **Difference (pH)** | **Average** | **Standard Deviation** |
| --- | --- | --- | --- |
| 0 | Sat. Paste – Top *in situ* | 0.59 | 0.39 |
|  | Pour-through – Bottom *in situ* | 0.45 | 0.40 |
|  | Sat. paste – Pour-through | 0.23 | 0.35 |
| 25 | Sat. Paste – Top *in situ* | 0.40 | 0.10 |
|  | Pour-through – Bottom *in situ* | 0.62 | 0.23 |
|  | Sat. paste – Pour-through | 0.12 | 0.19 |
| 50 | Sat. Paste – Top *in situ* | 0.64 | 0.12 |
|  | Pour-through – Bottom *in situ* | 0.13 | 0.17 |
|  | Sat. paste – Pour-through | 0.42 | 0.21 |
| 75 | Sat. Paste – Top *in situ* | 0.52 | 0.23 |
|  | Pour-through – Bottom *in situ* | 0.36 | 0.19 |
|  | Sat. paste – Pour-through | 0.05 | 0.11 |

**Supplementary Table 15**. The pH of unplanted coconut coir containers (n = 3) with increasing perlite volumes using the pour-through, saturated paste top *in situ*, and bottom *in situ* methods.

| **Perlite (%)** | **Pour-through** | **Saturated paste** | | |  | **Top *in situ*** | | |  | **Bottom *in situ*** | | |
| --- | --- | --- | --- | --- | --- | --- | --- | --- | --- | --- | --- | --- |
| 0 | 5.87 | 6.66 | 6.47 | 6.60 |  | 6.07 | 6.33 | 6.10 |  | 5.37 | 5.51 | 5.31 |
|  | 5.88 | 6.50 | 6.59 | 6.61 |  | 6.33 | 6.28 | 6.37 |  | 5.50 | 5.40 | 5.58 |
|  | 5.95 | 6.47 | 6.52 | 6.66 |  | 6.07 | 6.07 | 6.08 |  | 5.44 | 5.47 | 5.39 |
| 25 | 6.18 | 6.60 | 6.63 | 6.64 |  | 6.46 | 6.45 | 6.37 |  | 5.66 | 5.62 | 5.63 |
|  | 6.10 | 6.79 | 6.81 | 6.63 |  | 6.34 | 6.29 | 6.35 |  | 5.87 | 5.38 | 5.37 |
|  | 6.07 | 6.68 | 6.37 | 6.53 |  | 6.39 | 6.31 | 6.36 |  | 5.67 | 5.48 | 5.50 |
| 50 | 6.46 | 7.12 | 6.86 | 7.11 |  | 6.43 | 6.38 | 6.20 |  | 6.19 | 6.08 | 6.12 |
|  | 6.36 | 7.04 | 7.01 | 6.93 |  | 6.14 | 6.31 | 6.33 |  | 6.07 | 5.93 | 6.08 |
|  | 6.35 | 6.93 | 7.22 | 6.77 |  | 6.23 | 6.09 | 6.56 |  | 6.13 | 5.73 | 5.99 |
| 75 | 6.97 | 7.20 | 7.06 | 7.09 |  | 6.74 | 6.62 | 6.45 |  | 6.74 | 6.67 | 6.62 |
|  | 6.88 | 7.46 | 7.20 | 7.38 |  | 6.78 | 7.08 | 6.89 |  | 6.65 | 6.62 | 6.64 |
|  | 6.96 | 7.20 | 7.17 | 7.21 |  | 6.68 | 6.73 | 6.85 |  | 6.74 | 6.45 | 6.46 |

**Supplementary Table 16**. The average and standard deviation of pH in unplanted coconut coir containers (n = 3) with increasing perlite volumes using the pour-through, saturated paste top *in situ*, and bottom *in situ* methods.

| **Perlite (%)** | **Pour-through** | |  | **Saturated paste** | |  | **Top insertion** | |  | **Bottom insertion** | |
| --- | --- | --- | --- | --- | --- | --- | --- | --- | --- | --- | --- |
|  | **Avg.** | **S.Dev** |  | **Avg.** | **S.Dev** |  | **Avg.** | **S.Dev** |  | **Avg.** | **S.Dev** |
| 0 | 5.90 | 0.04 |  | 6.56 | 0.08 |  | 6.19 | 0.13 |  | 5.44 | 0.08 |
| 25 | 6.11 | 0.06 |  | 6.63 | 0.13 |  | 6.37 | 0.06 |  | 5.58 | 0.16 |
| 75 | 6.39 | 0.06 |  | 7.00 | 0.14 |  | 6.30 | 0.15 |  | 6.04 | 0.14 |
| 100 | 6.94 | 0.05 |  | 7.22 | 0.13 |  | 6.76 | 0.18 |  | 6.62 | 0.10 |

**Supplementary Table 17.** The average differences and standard deviations among pH measurement methods in unplanted coconut coir with increasing perlite volumes.

| **Perlite (%)** | **Difference (pH)** | **Average** | **Standard deviation** |
| --- | --- | --- | --- |
| 0 | Sat. Paste – Top *in situ* | 0.38 | 0.17 |
|  | Pour-through – Bottom *in situ* | 0.46 | 0.09 |
|  | Sat. paste – Pour-through | 0.66 | 0.09 |
| 25 | Sat. Paste – Top *in situ* | 0.26 | 0.15 |
|  | Pour-through – Bottom *in situ* | 0.54 | 0.15 |
|  | Sat. paste – Pour-through | 0.51 | 0.13 |
| 50 | Sat. Paste – Top *in situ* | 0.70 | 0.27 |
|  | Pour-through – Bottom *in situ* | 0.35 | 0.12 |
|  | Sat. paste – Pour-through | 0.61 | 0.14 |
| 75 | Sat. Paste – Top *in situ* | 0.46 | 0.16 |
|  | Pour-through – Bottom *in situ* | 0.32 | 0.12 |
|  | Sat. paste – Pour-through | 0.28 | 0.16 |

**Supplementary Table 18**. The pH of unplanted bark containers (n = 3) with increasing perlite volumes using the pour-through, saturated paste top *in situ*, and bottom *in situ* methods.

| **Perlite (%)** | **Pour-through** | **Saturated paste** | | |  | **Top *in situ*** | | |  | **Bottom *in situ*** | | |
| --- | --- | --- | --- | --- | --- | --- | --- | --- | --- | --- | --- | --- |
| 0 | 5.65 | 4.92 | 4.52 | 4.17 |  | 4.01 | 4.00 | 3.86 |  | 3.67 | 3.43 | 3.62 |
|  | 5.15 | 4.50 | 4.26 | 4.96 |  | 3.84 | 3.73 | 3.83 |  | 3.74 | 3.56 | 3.40 |
|  | 4.43 | 5.04 | 4.34 | 5.13 |  | 4.05 | 3.86 | 3.92 |  | 3.53 | 3.38 | 3.23 |
| 25 | 5.52 | 5.73 | 5.54 | 5.34 |  | 4.84 | 4.28 | 3.97 |  | 3.74 | 3.74 | 3.44 |
|  | 5.19 | 4.72 | 5.15 | 5.12 |  | 3.52 | 3.98 | 4.04 |  | 3.72 | 3.60 | 3.55 |
|  | 5.46 | 4.82 | 5.27 | 5.57 |  | 3.97 | 3.88 | 4.10 |  | 3.82 | 3.39 | 3.56 |
| 50 | 6.09 | 5.58 | 4.88 | 5.02 |  | 5.00 | 4.26 | 4.34 |  | 4.27 | 4.02 | 4.32 |
|  | 6.27 | 5.69 | 4.72 | 5.29 |  | 4.64 | 4.62 | 4.30 |  | 4.06 | 4.32 | 4.31 |
|  | 5.97 | 5.23 | 5.08 | 4.58 |  | 4.82 | 4.46 | 4.35 |  | 4.45 | 3.97 | 4.06 |
| 75 | 6.22 | 6.20 | 6.02 | 6.01 |  | 5.66 | 4.88 | 4.98 |  | 4.76 | 4.89 | 4.96 |
|  | 6.16 | 5.97 | 6.01 | 6.00 |  | 4.78 | 4.62 | 4.46 |  | 4.54 | 4.54 | 4.78 |
|  | 6.36 | 6.27 | 5.73 | 5.69 |  | 4.91 | 5.10 | 4.52 |  | 4.49 | 4.91 | 4.60 |

**Supplementary Table 19**. The average and standard deviation of pH in unplanted bark containers (n = 3) with increasing perlite volumes using the pour-through, saturated paste top *in situ*, and bottom *in situ* methods.

| **Perlite (%)** | **Pour-through** | |  | **Saturated paste** | |  | **Top insertion** | |  | **Bottom insertion** | |
| --- | --- | --- | --- | --- | --- | --- | --- | --- | --- | --- | --- |
|  | **Avg.** | **S.Dev** |  | **Avg.** | **S.Dev** |  | **Avg.** | **S.Dev** |  | **Avg.** | **S.Dev** |
| 0 | 5.08 | 0.61 |  | 4.65 | 0.37 |  | 3.90 | 0.10 |  | 3.51 | 0.16 |
| 25 | 5.39 | 0.18 |  | 5.14 | 0.38 |  | 4.06 | 0.35 |  | 3.62 | 0.15 |
| 75 | 6.11 | 0.15 |  | 5.12 | 0.37 |  | 4.53 | 0.26 |  | 4.20 | 0.17 |
| 100 | 6.25 | 0.10 |  | 5.99 | 0.19 |  | 4.88 | 0.36 |  | 4.72 | 0.18 |

**Supplementary Table 20.** The average differences and standard deviations among pH measurement methods in unplanted bark with increasing perlite volume.

| **Perlite (%)** | **Difference (pH)** | **Average** | **Standard Deviation** |
| --- | --- | --- | --- |
| 0 | Sat. Paste – Top *in situ* | 0.75 | 0.32 |
|  | Pour-through – Bottom *in situ* | 1.57 | 0.46 |
|  | Sat. paste – Pour-through | -0.43 | 0.37 |
| 25 | Sat. Paste – Top *in situ* | 1.08 | 0.29 |
|  | Pour-through – Bottom *in situ* | 1.77 | 0.21 |
|  | Sat. paste – Pour-through | -0.25 | 0.35 |
| 50 | Sat. Paste – Top *in situ* | 0.59 | 0.31 |
|  | Pour-through – Bottom *in situ* | 1.91 | 0.20 |
|  | Sat. paste – Pour-through | -0.99 | 0.35 |
| 75 | Sat. Paste – Top *in situ* | 1.11 | 0.34 |
|  | Pour-through – Bottom *in situ* | 1.53 | 0.20 |
|  | Sat. paste – Pour-through | -0.26 | 0.23 |

**Supplementary Table 21**. The pH of planted peat moss containers (n = 3) with increasing perlite volumes using the pour-through, saturated paste top *in situ*, and bottom *in situ* methods.

| **Perlite (%)** | **Pour-through** | **Saturated paste** | | |  | **Top *in situ*** | | |  | **Bottom *in situ*** | | |
| --- | --- | --- | --- | --- | --- | --- | --- | --- | --- | --- | --- | --- |
| 0 | 4.47 | 5.15 | 5.09 | 5.67 |  | 5.07 | 4.15 | 4.32 |  | 3.99 | 3.78 | 4.11 |
|  | 4.24 | 6.06 | 5.29 | 6.08 |  | 4.55 | 4.67 | 4.69 |  | 3.91 | 3.85 | 3.78 |
|  | 4.50 | 5.23 | 5.61 | 5.68 |  | 4.71 | 4.79 | 4.04 |  | 3.87 | 3.88 | 3.92 |
| 25 | 4.42 | 5.51 | 5.58 | 4.83 |  | 4.56 | 4.99 | 4.41 |  | 3.99 | 3.94 | 3.84 |
|  | 4.37 | 5.22 | 5.30 | 5.26 |  | 4.94 | 4.54 | 4.49 |  | 3.95 | 3.92 | 3.92 |
|  | 4.50 | 4.97 | 5.35 | 5.47 |  | 4.41 | 4.84 | 4.90 |  | 3.96 | 3.81 | 4.01 |
| 50 | 5.17 | 5.74 | 4.90 | 5.60 |  | 5.16 | 3.82 | 4.79 |  | 4.16 | 3.94 | 4.29 |
|  | 5.00 | 5.31 | 5.56 | 5.81 |  | 4.72 | 5.05 | 5.10 |  | 4.26 | 4.10 | 4.45 |
|  | 5.08 | 6.05 | 5.77 | 5.93 |  | 5.18 | 5.22 | 4.83 |  | 3.99 | 4.17 | 4.04 |
| 75 | 6.41 | 6.35 | 5.81 | 5.94 |  | 5.29 | 4.71 | 5.19 |  | 4.59 | 4.56 | 4.83 |
|  | 6.75 | 6.32 | 6.25 | 6.24 |  | 5.72 | 6.04 | 5.17 |  | 4.49 | 4.57 | 4.55 |
|  | 6.31 | 6.17 | 5.85 | 6.12 |  | 5.75 | 5.17 | 6.11 |  | 4.49 | 4.24 | 4.41 |

**Supplementary Table 22**. The average and standard deviation of pH in planted peat moss containers (n = 3) with increasing perlite volumes using the pour-through, saturated paste top *in situ*, and bottom *in situ* methods.

| **Perlite (%)** | **Pour-through** | |  | **Saturated paste** | |  | **Top insertion** | |  | **Bottom insertion** | |
| --- | --- | --- | --- | --- | --- | --- | --- | --- | --- | --- | --- |
|  | **Avg.** | **S.Dev** |  | **Avg.** | **S.Dev** |  | **Avg.** | **S.Dev** |  | **Avg.** | **S.Dev** |
| 0 | 4.40 | 0.14 |  | 5.54 | 0.37 |  | 4.55 | 0.33 |  | 3.90 | 0.10 |
| 25 | 4.43 | 0.07 |  | 5.28 | 0.25 |  | 4.68 | 0.24 |  | 3.93 | 0.07 |
| 75 | 5.08 | 0.09 |  | 5.63 | 0.35 |  | 4.87 | 0.44 |  | 4.16 | 0.16 |
| 100 | 6.49 | 0.23 |  | 6.12 | 0.20 |  | 5.46 | 0.47 |  | 4.53 | 0.16 |

**Supplementary Table 23.** The average differences and standard deviations among pH measurement methods in planted peat moss with increasing perlite volume.

| **Perlite (%)** | **Difference (pH)** | **Average** | **Standard Deviation** |
| --- | --- | --- | --- |
| 0 | Sat. Paste – Top *in situ* | 0.99 | 0.52 |
|  | Pour-through – Bottom *in situ* | 0.50 | 0.13 |
|  | Sat. paste – Pour-through | 1.14 | 0.45 |
| 25 | Sat. Paste – Top *in situ* | 0.60 | 0.20 |
|  | Pour-through – Bottom *in situ* | 0.50 | 0.09 |
|  | Sat. paste – Pour-through | 0.85 | 0.25 |
| 50 | Sat. Paste – Top *in situ* | 0.76 | 0.22 |
|  | Pour-through – Bottom *in situ* | 0.93 | 0.20 |
|  | Sat. paste – Pour-through | 0.55 | 0.37 |
| 75 | Sat. Paste – Top *in situ* | 0.66 | 0.39 |
|  | Pour-through – Bottom *in situ* | 1.96 | 0.23 |
|  | Sat. paste – Pour-through | -0.37 | 0.19 |

**Supplementary Table 24**. The pH of planted peat moss with lime amendment of 0.4 g hydrated lime per kg (n = 3) with increasing perlite volumes using the pour-through, saturated paste top *in situ*, and bottom *in situ* methods.

| **Perlite (%)** | **Pour-through** | **Saturated paste** | | |  | **Top *in situ*** | | |  | **Bottom *in situ*** | | |
| --- | --- | --- | --- | --- | --- | --- | --- | --- | --- | --- | --- | --- |
| 0 | 4.54 | 5.86 | 5.32 | 5.11 |  | 4.84 | 4.70 | 4.51 |  | 4.07 | 4.12 | 4.23 |
|  | 4.55 | 4.65 | 4.82 | 5.45 |  | 4.75 | 4.20 | 4.56 |  | 4.35 | 4.19 | 4.23 |
|  | 4.55 | 5.39 | 5.61 | 5.86 |  | 4.49 | 4.45 | 4.81 |  | 4.30 | 4.18 | 4.26 |
| 25 | 5.06 | 5.96 | 5.66 | 5.92 |  | 5.30 | 4.79 | 4.44 |  | 4.43 | 4.41 | 4.15 |
|  | 4.60 | 5.48 | 6.15 | 6.00 |  | 4.64 | 4.65 | 4.83 |  | 4.40 | 4.11 | 4.20 |
|  | 4.89 | 6.09 | 5.68 | 5.83 |  | 4.76 | 4.34 | 3.74 |  | 4.30 | 4.22 | 4.38 |
| 50 | 5.66 | 6.08 | 6.06 | 6.30 |  | 5.41 | 5.28 | 5.58 |  | 4.59 | 4.92 | 4.81 |
|  | 4.73 | 6.54 | 6.33 | 6.44 |  | 5.77 | 5.53 | 5.45 |  | 5.13 | 4.66 | 4.30 |
|  | 4.93 | 5.78 | 6.35 | 6.06 |  | 4.90 | 5.39 | 5.38 |  | 4.32 | 4.34 | 4.41 |
| 75 | 6.33 | 6.20 | 6.41 | 6.54 |  | 6.18 | 6.06 | 5.97 |  | 5.18 | 5.54 | 5.20 |
|  | 6.27 | 6.22 | 6.35 | 6.31 |  | 5.69 | 5.78 | 5.82 |  | 5.55 | 5.65 | 5.60 |
|  | 6.12 | 6.19 | 6.09 | 6.22 |  | 5.63 | 5.36 | 5.75 |  | 4.90 | 5.29 | 5.03 |

**Supplementary Table 25**. The average and standard deviation of pH in planted peat moss with lime amendment of 0.4 g hydrated lime per kg containers (n = 3) with increasing perlite volumes using the pour-through, saturated paste top *in situ*, and bottom *in situ* methods.

| **Perlite (%)** | **Pour-through** | |  | **Saturated paste** | |  | **Top insertion** | |  | **Bottom insertion** | |
| --- | --- | --- | --- | --- | --- | --- | --- | --- | --- | --- | --- |
|  | **Avg.** | **S.Dev** |  | **Avg.** | **S.Dev** |  | **Avg.** | **S.Dev** |  | **Avg.** | **S.Dev** |
| 0 | 4.55 | 0.01 |  | 5.34 | 0.42 |  | 4.59 | 0.21 |  | 4.21 | 0.09 |
| 25 | 4.85 | 0.23 |  | 5.86 | 0.22 |  | 4.61 | 0.42 |  | 4.29 | 0.12 |
| 75 | 5.11 | 0.49 |  | 6.22 | 0.24 |  | 5.41 | 0.24 |  | 4.61 | 0.30 |
| 100 | 6.24 | 0.11 |  | 6.28 | 0.14 |  | 5.80 | 0.24 |  | 5.33 | 0.27 |

**Supplementary Table 26.** The difference in multiple methods of measuring pH values in planted peat with lime amendment of 0.4 g hydrated lime per kg peat at increasing levels of perlite.

| **Perlite (%)** | **Difference (pH)** | **Average** | **Standard Deviation** |
| --- | --- | --- | --- |
| 0 | Sat. Paste – Top *in situ* | 0.75 | 0.38 |
|  | Pour-through – Bottom *in situ* | 0.33 | 0.08 |
|  | Sat. paste – Pour-through | 0.79 | 0.42 |
| 25 | Sat. Paste – Top *in situ* | 1.25 | 0.43 |
|  | Pour-through – Bottom *in situ* | 0.56 | 0.20 |
|  | Sat. paste – Pour-through | 1.01 | 0.31 |
| 50 | Sat. Paste – Top *in situ* | 0.81 | 0.12 |
|  | Pour-through – Bottom *in situ* | 0.50 | 0.44 |
|  | Sat. paste – Pour-through | 1.11 | 0.55 |
| 75 | Sat. Paste – Top *in situ* | 0.48 | 0.20 |
|  | Pour-through – Bottom *in situ* | 0.91 | 0.23 |
|  | Sat. paste – Pour-through | 0.04 | 0.10 |

**Supplementary Table 27**. The pH of planted peat moss with lime amendment of 0.9 g hydrated lime per L (n = 3) with increasing perlite volumes using the pour-through, saturated paste top *in situ*, and bottom *in situ* methods.

| **Perlite (%)** | **Pour-through** | **Saturated paste** | | |  | **Top *in situ*** | | |  | **Bottom *in situ*** | | |
| --- | --- | --- | --- | --- | --- | --- | --- | --- | --- | --- | --- | --- |
| 0 | 5.83 | 6.11 | 5.71 | 5.55 |  | 5.48 | 5.15 | 5.19 |  | 4.74 | 4.91 | 5.03 |
|  | 5.23 | 5.95 | 5.58 | 6.08 |  | 5.38 | 4.97 | 5.16 |  | 4.78 | 4.64 | 5.15 |
|  | 4.96 | 6.16 | 5.44 | 6.42 |  | 4.47 | 5.00 | 5.38 |  | 4.59 | 4.77 | 4.66 |
| 25 | 5.57 | 5.90 | 5.79 | 6.41 |  | 5.13 | 5.13 | 5.62 |  | 4.60 | 5.45 | 4.68 |
|  | 5.00 | 6.17 | 6.00 | 6.12 |  | 5.17 | 5.26 | 5.54 |  | 4.80 | 4.68 | 5.01 |
|  | 5.44 | 6.05 | 6.02 | 5.99 |  | 5.15 | 5.13 | 5.34 |  | 4.77 | 4.46 | 4.66 |
| 50 | 5.76 | 6.43 | 6.29 | 6.40 |  | 5.69 | 5.50 | 5.70 |  | 5.00 | 4.85 | 5.05 |
|  | 5.55 | 6.19 | 6.26 | 6.40 |  | 5.75 | 5.51 | 5.34 |  | 4.77 | 4.85 | 5.04 |
|  | 5.40 | 6.33 | 6.03 | 6.56 |  | 5.63 | 5.53 | 5.45 |  | 5.15 | 4.94 | 5.04 |
| 75 | 6.33 | 6.43 | 6.29 | 6.33 |  | 5.74 | 6.00 | 6.00 |  | 5.97 | 5.79 | 5.46 |
|  | 6.40 | 6.19 | 6.31 | 6.25 |  | 5.82 | 5.87 | 6.01 |  | 5.86 | 5.83 | 5.40 |
|  | 6.38 | 6.24 | 6.21 | 6.44 |  | 5.80 | 5.98 | 6.09 |  | 5.67 | 6.01 | 5.65 |

**Supplementary Table 28**. The average and standard deviation of pH in planted peat moss with lime amendment of 0.9 g hydrated lime per L containers (n = 3) with increasing perlite volumes using the pour-through, saturated paste top *in situ*, and bottom *in situ* methods.

| **Perlite (%)** | **Pour-through** | |  | **Saturated paste** | |  | **Top insertion** | |  | **Bottom insertion** | |
| --- | --- | --- | --- | --- | --- | --- | --- | --- | --- | --- | --- |
|  | **Avg.** | **S.Dev** |  | **Avg.** | **S.Dev** |  | **Avg.** | **S.Dev** |  | **Avg.** | **S.Dev** |
| 0 | 5.34 | 0.45 |  | 5.89 | 0.33 |  | 5.13 | 0.30 |  | 4.81 | 0.19 |
| 25 | 5.34 | 0.30 |  | 6.06 | 0.18 |  | 5.27 | 0.19 |  | 4.79 | 0.29 |
| 75 | 5.57 | 0.18 |  | 6.32 | 0.15 |  | 5.57 | 0.13 |  | 4.97 | 0.12 |
| 100 | 6.37 | 0.04 |  | 6.30 | 0.09 |  | 5.92 | 0.12 |  | 5.74 | 0.21 |

**Supplementary Table 29.** The difference in multiple methods of measuring pH values in planted peat with lime amendment of 0.9 g hydrated lime per L (n = 3) at increasing levels of perlite.

| **Perlite (%)** | **Difference (pH)** | **Average** | **Standard Deviation** |
| --- | --- | --- | --- |
| 0 | Sat. Paste – Top *in situ* | 0.76 | 0.41 |
|  | Pour-through – Bottom *in situ* | 0.53 | 0.34 |
|  | Sat. paste – Pour-through | 0.55 | 0.57 |
| 25 | Sat. Paste – Top *in situ* | 0.78 | 0.12 |
|  | Pour-through – Bottom *in situ* | 0.55 | 0.39 |
|  | Sat. paste – Pour-through | 0.72 | 0.35 |
| 50 | Sat. Paste – Top *in situ* | 0.75 | 0.22 |
|  | Pour-through – Bottom *in situ* | 0.60 | 0.22 |
|  | Sat. paste – Pour-through | 0.75 | 0.20 |
| 75 | Sat. Paste – Top *in situ* | 0.38 | 0.14 |
|  | Pour-through – Bottom *in situ* | 0.63 | 0.22 |
|  | Sat. paste – Pour-through | -0.07 | 0.11 |

**Supplementary Table 30**. The pH of planted peat with lime amendment of 4.4 g hydrated lime per L (n = 3) with increasing perlite volumes using the pour-through, saturated paste top *in situ*, and bottom *in situ* methods.

| **Perlite (%)** | **Pour-through** | **Saturated paste** | | |  | **Top *in situ*** | | |  | **Bottom *in situ*** | | |
| --- | --- | --- | --- | --- | --- | --- | --- | --- | --- | --- | --- | --- |
| 0 | 6.50 | 6.88 | 7.06 | 6.92 |  | 6.48 | 6.22 | 6.28 |  | 6.41 | 6.24 | 5.75 |
|  | 6.68 | 6.57 | 6.79 | 6.67 |  | 6.23 | 6.57 | 6.06 |  | 6.68 | 5.95 | 6.17 |
|  | 6.71 | 6.42 | 6.54 | 6.92 |  | 6.35 | 6.16 | 6.50 |  | 6.14 | 6.46 | 6.42 |
| 25 | 6.99 | 6.81 | 6.74 | 6.83 |  | 6.80 | 6.84 | 6.95 |  | 7.03 | 6.60 | 6.29 |
|  | 6.93 | 6.87 | 6.63 | 6.65 |  | 6.15 | 6.48 | 6.32 |  | 6.60 | 6.64 | 6.68 |
|  | 6.81 | 6.94 | 6.86 | 6.82 |  | 6.51 | 6.63 | 6.61 |  | 6.55 | 6.33 | 6.64 |
| 50 | 6.76 | 6.78 | 6.52 | 6.57 |  | 6.67 | 6.46 | 6.50 |  | 6.44 | 6.68 | 6.61 |
|  | 6.91 | 6.81 | 7.00 | 5.83 |  | 6.63 | 6.66 | 6.35 |  | 6.67 | 6.47 | 6.67 |
|  | 6.80 | 6.60 | 6.76 | 6.32 |  | 6.32 | 6.72 | 6.33 |  | 6.72 | 6.75 | 5.83 |
| 75 | 6.86 | 6.59 | 6.82 | 6.66 |  | 6.45 | 6.39 | 6.72 |  | 6.65 | 6.78 | 6.53 |
|  | 6.87 | 6.87 | 6.59 | 6.51 |  | 6.76 | 6.60 | 6.00 |  | 6.80 | 6.71 | 6.79 |
|  | 6.85 | 6.61 | 6.62 | 6.60 |  | 6.48 | 6.40 | 6.36 |  | 6.83 | 6.59 | 6.41 |

**Supplementary Table 31**. The average and standard deviation of pH in planted peat with lime amendment of 4.4 g hydrated lime per L (n = 3) with increasing perlite volumes using the pour-through, saturated paste top *in situ*, and bottom *in situ* methods

| **Perlite (%)** | **Pour-through** | |  | **Saturated paste** | |  | **Top insertion** | |  | **Bottom insertion** | |
| --- | --- | --- | --- | --- | --- | --- | --- | --- | --- | --- | --- |
|  | **Avg.** | **S.Dev** |  | **Avg.** | **S.Dev** |  | **Avg.** | **S.Dev** |  | **Avg.** | **S.Dev** |
| 0 | 6.63 | 0.11 |  | 6.75 | 0.21 |  | 6.32 | 0.17 |  | 6.25 | 0.28 |
| 25 | 6.91 | 0.09 |  | 6.79 | 0.10 |  | 6.59 | 0.26 |  | 6.60 | 0.21 |
| 75 | 6.82 | 0.08 |  | 6.58 | 0.34 |  | 6.52 | 0.16 |  | 6.54 | 0.29 |
| 100 | 6.86 | 0.01 |  | 6.65 | 0.12 |  | 6.46 | 0.23 |  | 0.68 | 0.14 |

**Supplementary Table 32.** The average differences and standard deviations among pH measurement methods in planted peat with lime amendment of 4.4 g hydrated lime per L (n = 3) with increasing perlite volumes.

| **Perlite (%)** | **Difference (pH)** | **Average** | **Standard Deviation** |
| --- | --- | --- | --- |
| 0 | Sat. Paste – Top *in situ* | 0.44 | 0.23 |
|  | Pour-through – Bottom *in situ* | 0.38 | 0.27 |
|  | Sat. paste – Pour-through | 0.12 | 0.29 |
| 25 | Sat. Paste – Top *in situ* | 0.21 | 0.27 |
|  | Pour-through – Bottom *in situ* | 0.31 | 0.21 |
|  | Sat. paste – Pour-through | -0.12 | 0.15 |
| 50 | Sat. Paste – Top *in situ* | 0.06 | 0.25 |
|  | Pour-through – Bottom *in situ* | 0.29 | 0.29 |
|  | Sat. paste – Pour-through | -0.25 | 0.35 |
| 75 | Sat. Paste – Top *in situ* | 0.19 | 0.19 |
|  | Pour-through – Bottom *in situ* | 0.18 | 0.14 |
|  | Sat. paste – Pour-through | -0.21 | 0.11 |

**Supplementary Table 33**. The pH of planted coconut coir containers (n = 3) with increasing perlite volumes using the pour-through, saturated paste top *in situ*, and bottom *in situ* methods.

| **Perlite (%)** | **Pour-through** | **Saturated paste** | | |  | **Top *in situ*** | | |  | **Bottom *in situ*** | | |
| --- | --- | --- | --- | --- | --- | --- | --- | --- | --- | --- | --- | --- |
| 0 | 6.34 | 6.51 | 6.54 | 6.47 |  | 6.19 | 6.31 | 6.14 |  | 6.23 | 6.26 | 6.15 |
|  | 6.38 | 6.32 | 6.41 | 6.26 |  | 6.18 | 6.28 | 6.20 |  | 6.24 | 6.20 | 6.17 |
|  | 6.54 | 6.72 | 6.59 | 6.63 |  | 6.20 | 6.12 | 6.41 |  | 6.30 | 6.38 | 6.29 |
| 25 | 6.43 | 6.71 | 6.74 | 6.63 |  | 6.07 | 6.49 | 6.13 |  | 6.32 | 6.27 | 6.13 |
|  | 6.44 | 6.69 | 6.74 | 6.74 |  | 6.13 | 6.42 | 6.47 |  | 6.33 | 6.13 | 6.19 |
|  | 6.48 | 6.74 | 6.68 | 6.67 |  | 6.21 | 6.23 | 6.11 |  | 6.26 | 6.15 | 6.16 |
| 50 | 6.47 | 6.43 | 6.55 | 6.62 |  | 5.90 | 6.26 | 6.47 |  | 6.19 | 6.18 | 6.11 |
|  | 6.50 | 6.55 | 6.54 | 6.52 |  | 6.25 | 6.28 | 6.28 |  | 6.24 | 6.26 | 6.05 |
|  | 6.50 | 6.74 | 6.63 | 6.43 |  | 6.48 | 6.38 | 6.37 |  | 6.00 | 6.12 | 6.12 |
| 75 | 6.58 | 6.55 | 6.45 | 6.59 |  | 6.04 | 6.03 | 6.56 |  | 6.25 | 6.40 | 6.37 |
|  | 6.61 | 6.82 | 6.66 | 6.57 |  | 6.49 | 6.41 | 6.37 |  | 6.33 | 6.04 | 6.28 |
|  | 6.63 | 6.81 | 6.66 | 6.65 |  | 6.72 | 6.54 | 6.20 |  | 6.11 | 6.40 | 6.44 |

**Supplementary Table 34**. The average and standard deviation of pH in planted coconut coir containers (n = 3) with increasing perlite volumes using the pour-through, saturated paste top *in situ*, and bottom *in situ* methods.

| **Perlite (%)** | **Pour-through** | |  | **Saturated paste** | |  | **Top insertion** | |  | **Bottom insertion** | |
| --- | --- | --- | --- | --- | --- | --- | --- | --- | --- | --- | --- |
|  | **Avg.** | **S.Dev** |  | **Avg.** | **S.Dev** |  | **Avg.** | **S.Dev** |  | **Avg.** | **S.Dev** |
| 0 | 6.42 | 0.11 |  | 6.49 | 0.15 |  | 6.23 | 0.09 |  | 6.25 | 0.07 |
| 25 | 6.45 | 0.03 |  | 6.70 | 0.04 |  | 6.25 | 0.16 |  | 6.22 | 0.08 |
| 75 | 6.49 | 0.02 |  | 6.56 | 0.10 |  | 6.30 | 0.17 |  | 6.14 | 0.09 |
| 100 | 6.61 | 0.03 |  | 6.64 | 0.12 |  | 6.37 | 0.24 |  | 6.29 | 0.14 |

**Supplementary Table 35.** The average differences and standard deviations among pH measurement methods in planted coconut coir with increasing perlite volumes.

| **Perlite (%)** | **Difference (pH)** | **Average** | **Standard Deviation** |
| --- | --- | --- | --- |
| 0 | Sat. Paste – Top *in situ* | 0.27 | 0.16 |
|  | Pour-through – Bottom *in situ* | 0.17 | 0.06 |
|  | Sat. paste – Pour-through | 0.07 | 0.11 |
| 25 | Sat. Paste – Top *in situ* | 0.45 | 0.14 |
|  | Pour-through – Bottom *in situ* | 0.23 | 0.09 |
|  | Sat. paste – Pour-through | 0.25 | 0.05 |
| 50 | Sat. Paste – Top *in situ* | 0.26 | 0.13 |
|  | Pour-through – Bottom *in situ* | 0.35 | 0.09 |
|  | Sat. paste – Pour-through | 0.07 | 0.10 |
| 75 | Sat. Paste – Top *in situ* | 0.27 | 0.17 |
|  | Pour-through – Bottom *in situ* | 0.32 | 0.14 |
|  | Sat. paste – Pour-through | 0.03 | 0.11 |

**Supplementary Table 36**. The pH of planted bark containers (n = 3) with increasing perlite volumes using the pour-through, saturated paste top *in situ*, and bottom *in situ* methods.

| **Perlite (%)** | **Pour-through** | **Saturated paste** | | |  | **Top *in situ*** | | |  | **Bottom *in situ*** | | |
| --- | --- | --- | --- | --- | --- | --- | --- | --- | --- | --- | --- | --- |
| 0 | 4.61 | 4.81 | 4.62 | 4.13 |  | 4.81 | 4.62 | 4.13 |  | 5.03 | 4.50 | 4.02 |
|  | 5.22 | 4.58 | 4.39 | 4.49 |  | 4.14 | 4.26 | 4.19 |  | 3.81 | 3.89 | 3.87 |
|  | 4.51 | 4.59 | 4.31 | 4.64 |  | 4.16 | 4.10 | 4.19 |  | 3.96 | 3.83 | 4.06 |
| 25 | 5.54 | 4.53 | 4.65 | 4.66 |  | 4.21 | 4.37 | 4.32 |  | 4.30 | 4.13 | 4.22 |
|  | 5.60 | 4.75 | 4.93 | 4.73 |  | 4.37 | 4.54 | 4.20 |  | 4.16 | 4.24 | 4.12 |
|  | 5.58 | 4.82 | 4.89 | 4.98 |  | 4.29 | 4.45 | 4.49 |  | 4.29 | 4.33 | 3.96 |
| 50 | 6.22 | 4.76 | 4.94 | 4.99 |  | 4.66 | 4.47 | 4.81 |  | 4.84 | 4.83 | 4.56 |
|  | 6.32 | 4.73 | 4.99 | 4.86 |  | 4.47 | 4.56 | 4.72 |  | 5.05 | 4.36 | 4.42 |
|  | 6.23 | 5.08 | 5.03 | 4.88 |  | 4.91 | 4.51 | 4.66 |  | 4.83 | 4.57 | 4.46 |
| 75 | 6.31 | 5.18 | 5.57 | 5.19 |  | 5.23 | 5.01 | 5.17 |  | 4.76 | 4.77 | 4.60 |
|  | 6.30 | 5.12 | 5.61 | 5.39 |  | 4.79 | 5.38 | 5.07 |  | 4.65 | 4.98 | 5.13 |
|  | 6.39 | 5.01 | 5.31 | 5.20 |  | 4.74 | 5.78 | 4.89 |  | 5.85 | 4.41 | 5.50 |

**Supplementary Table 37**. The average and standard deviation of pH in bark coir containers (n = 3) with increasing perlite volumes using the pour-through, saturated paste top *in situ*, and bottom *in situ* methods.

| **Perlite (%)** | **Pour-through** | |  | **Saturated paste** | |  | **Top insertion** | |  | **Bottom insertion** | |
| --- | --- | --- | --- | --- | --- | --- | --- | --- | --- | --- | --- |
|  | **Avg.** | **S.Dev** |  | **Avg.** | **S.Dev** |  | **Avg.** | **S.Dev** |  | **Avg.** | **S.Dev** |
| 0 | 4.78 | 0.38 |  | 4.51 | 0.21 |  | 4.29 | 0.31 |  | 3.91 | 0.12 |
| 25 | 5.57 | 0.03 |  | 4.77 | 0.15 |  | 4.36 | 0.12 |  | 4.19 | 0.12 |
| 75 | 6.26 | 0.06 |  | 4.92 | 0.12 |  | 4.64 | 0.15 |  | 4.66 | 0.24 |
| 100 | 6.33 | 0.05 |  | 5.29 | 0.20 |  | 5.12 | 0.32 |  | 4.96 | 0.46 |

**Supplementary Table 38.** The average differences and standard deviations among pH measurement methods in planted bark with increasing perlite volumes.

| **Perlite (%)** | **Difference (pH)** | **Average** | **Standard Deviation** |
| --- | --- | --- | --- |
| 0 | Sat. Paste – Top *in situ* | 0.22 | 0.22 |
|  | Pour-through – Bottom *in situ* | 0.87 | 0.39 |
|  | Sat. paste – Pour-through | -0.27 | 0.41 |
| 25 | Sat. Paste – Top *in situ* | 0.41 | 0.09 |
|  | Pour-through – Bottom *in situ* | 1.38 | 0.12 |
|  | Sat. paste – Pour-through | -0.80 | 0.13 |
| 50 | Sat. Paste – Top *in situ* | 0.28 | 0.16 |
|  | Pour-through – Bottom *in situ* | 1.60 | 0.25 |
|  | Sat. paste – Pour-through | -1.34 | 0.14 |
| 75 | Sat. Paste – Top *in situ* | 0.17 | 0.30 |
|  | Pour-through – Bottom *in situ* | 1.37 | 0.45 |
|  | Sat. paste – Pour-through | -1.05 | 0.22 |

# Supplementary Figures


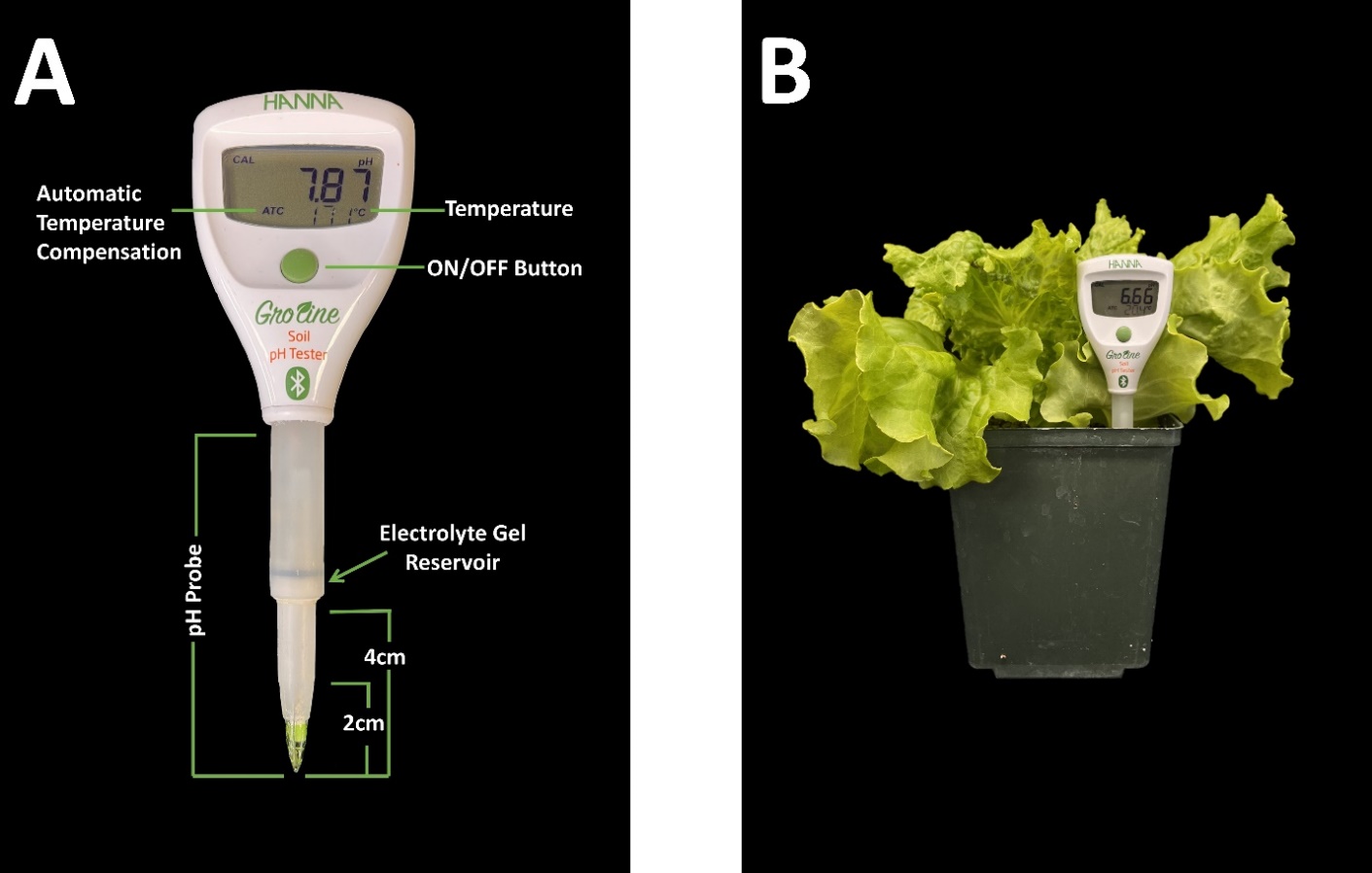


**Supplementary Figure 1.** The *in situ* pH meter used in this study. (A) A close-up view of the meter showing the pointed tip electrode. (B) The meter measuring the pH of a peat-based soilless media planted with lettuce (*Lactuca sativa* cv. Grand Rapids).


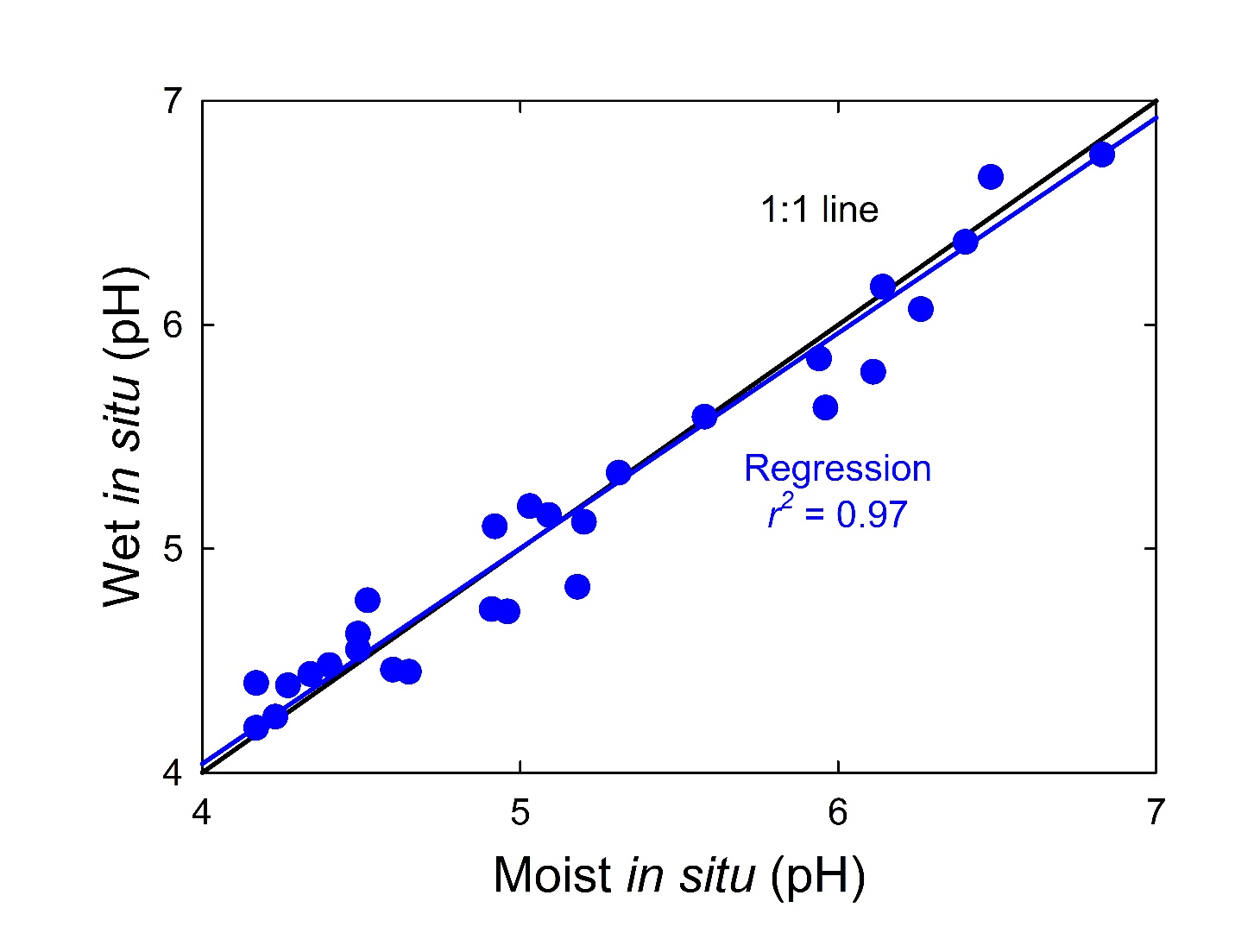


**Supplementary Figure 2.** The wet insertion *in situ* pH compared to the moist insertion *in situ* pH measured in 100% peat moss amended with increasing levels of hydrated lime. The black line indicates a perfect 1:1 relationship and the blue line indicates the regression line for the data, r^2^ = 0.97. All measurements were taken at a 4 cm depth.
